# Slipped Upper Femoral Epiphysis in Adolescents: Evolving Information on Its Mode of Presentation and Management

**DOI:** 10.7759/cureus.37674

**Published:** 2023-04-17

**Authors:** Annis Maatough, Meave Leonard, Hany Elbardesy, Satish Kutty

**Affiliations:** 1 Trauma and Orthopaedics, Sligo University Hospital, Sligo, IRL; 2 Trauma and Orthopaedics, East Kent University Hospitals NHS Foundation Trust, Kent, GBR

**Keywords:** management, paediatric orthopedics, adolescents., hip joint, sufe

## Abstract

Background and objective

Slipped upper femoral epiphysis (SUFE) is one of the most common hip pathologies in adolescents and pre-adolescents, the diagnosis of which is often missed due to delayed presentations. In this study, we aimed to conduct a retrospective analysis of SUFE cases treated in the hospital during the 15-year period from 2003 to 2018 and examine its bilateral presentation and the need for prophylactic pinning on the unaffected side.

Methods

This retrospective cohort study involved cases that were treated from 2003 to 2018. The case details were retrieved from the medical records department. Records older than 15 years were excluded owing to their inaccuracy, and 26 cases of SUFE were included in the final analysis. Each case was subjected to physical and radiological examinations of the symptomatic and asymptomatic hips. IBM SPSS Statistics v23 (IBM Corp., Armonk, NY) was used for data analysis.

Results

In this study, six of the 26 patients had bilateral SUFE and required subsequent surgical pinning. The duration of surgical interventions ranged from two to 22 months, while the mean intervention duration was 10.3 months. Among the cases, 61.5% (p<0.05) were idiopathic in nature upon documentation. However, 19% (p<0.05) of the cases were shown to be associated with an underlying condition or prior symptoms of the condition, whereas 7.6% (p<0.05) had an increased basal metabolic index; 11% (p<0.05) of the cases had an inherited family history of SUFE. A comparison between males and females showed a slightly higher frequency of complications in males (n=14) than in females (n=12) (p=0.556). The age of the patients at the presentation ranged between and 10-15 years, with an average age of 12.5 years.

Conclusion

Based on our findings, males were affected more than females and most of the cases were idiopathic. There is no significant evidence to support the need for prophylactic pinning of the unaffected hip. We recommend prospective studies with a larger sample of patients to gain more insight into the topic.

## Introduction

Slipped capital femoral epiphysis (SCFE), also called slipped upper femoral epiphysis (SUFE), is one of the most common hip pathologies in adolescents and pre-adolescents [1-3]. The incidence of SUFE ranges from 0.33/100,000 to 24.58/100,000 in children aged 8-15 years, and it has a male predominance [4,5]. The diagnosis of SUFE is often difficult because of the missed or delayed presentation, which can be due to the chronic nature of the presentation or atypical presentations such as knee or thigh pain. According to Novais and Millis, obesity is a risk factor for the development of SUFE, with long-term conditions depending on the severity of the deformity of the residual hip and the concomitant occurrence of complications such as avascular necrosis [6,7]. The management of SUFE has been a matter of various controversies, related to various classifications of the condition and the surgical operations that underpin the treatment of the condition [8]. Baig et al. [9] have posited that the presentation of hip pain and limping may be a deceptive form of contralateral hip pain or knee pain that can result in a misdiagnosis of the disease. The pathophysiology of the disease is not well understood, and both mechanical and hormonal factors are associated with SUFE [10].

Most of the classifications of SUFE are based on symptom duration. An acute slip could result in symptoms that could last less than three weeks, while the progression into chronic hip can take as long as several weeks or months [11]. Loder and Schneble have highlighted the significance of physical stability during the presentation of the disease by correlating it with the outcome of the disease [12]. From this perspective, a slip may be considered stable if walking is possible with or without crutches. Nonetheless, if walking is impossible even with crutches, the patient is regarded to have an unstable hip. The unaffected side of the hips is often subjected to prophylactic treatment, which can affect patients' well-being. Therefore, there is a need to examine if prophylactic pinning of the unaffected side is actually required. In this study, we aimed to conduct a retrospective study of SUFE cases treated in the hospital during the 15-year period from 2003 to 2018 and analyze its bilateral presentation and the need for prophylactic pinning on the unaffected side.

## Materials and methods

This was a retrospective cohort study involving patients with SUFE treated at the Sligo University Hospital (SUH) during a period of 15 years from 2003 to 2018. It was approved by the institutional review board of the hospital where the surgery was performed (approval number: 2019-234).

The case details of these patients were obtained from the medical records department and the Hospital Inpatient Enquiry (HIPE) office. Due to the limitations of technology in the past decade, records of cases beyond 15 years were not included owing to their inaccuracy. Obtaining the physical records of the cases was problematic due to the fact that the copies of the plain films were not stored. Moreover, the radiology software of the hospital did not keep records of some of the digitalized image data dated beyond 10 to 15 years, thus making the collection of data problematic. The images or hard copies of CT, MRI, and X-rays were obtained from outside the medical records department facilities.

Following the collection of patient records, there were 34 total cases of SUFE. However, based on our inclusion criteria focusing on patients with bilateral affection, eight of the cases were excluded and 26 cases were included in the final analysis. Each case was subjected to physical and radiological examinations of the symptomatic (hip pain and/or knee pain) and asymptomatic hips. The total number of hips was 52, and any of the cases that presented significant contralateral hip pathology either currently or subsequently were also documented. Every case was subjected to SUFE grading according to the degree of angulation, bilaterality, stability, severity, duration, and possible risk-posing capability. Moreover, any underlying pathology in terms of risk factors, follow-up, or complications to the overall outcome was also noted for each case. 

Ethical approval for conducting the study was obtained from the hospital ethics committee before commencing data collection. Caution was taken to ensure adherence to the recommendations and instructions given by the ethical committee during the course of the study. All data were analyzed using IBM SPSS Statistics v23 (IBM Corp., Armonk, NY) [13]. Qualitative variables were presented as frequencies and percentages, while mean and standard deviation (SD) were used to present quantitative variables.

## Results

**Figure 1:**
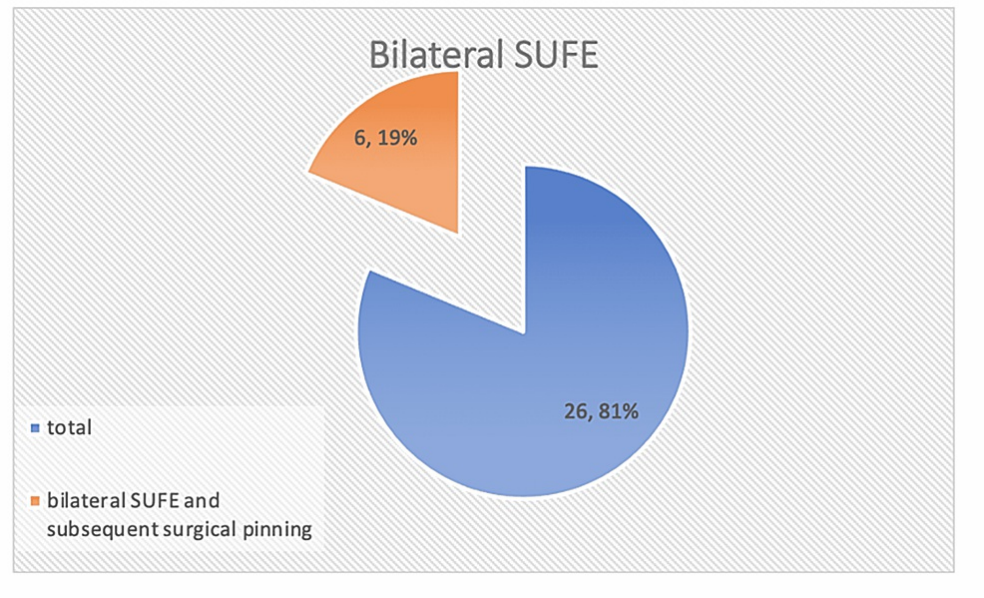
Pie chart displaying the proportion of bilateral cases out of total cases SUFE: slipped upper femoral epiphysis

The analysis was based on 26 hospital charts, which detailed confirmed cases of the SUFE that had been treated at Sligo University Hospital. These patients had undergone treatment from 2003 to 2018. The included cases had undergone in situ pinning treatment using either two screws or a single screw. An analysis of the results confirmed that six of the 26 patients developed bilateral SUFE and required subsequent surgical pinning (Figure 1). The duration of surgical interventions ranged from two to 22 months, while the mean intervention duration was 10.3 months.

Moreover, an analysis of the results indicated that one patient had prophylactic pinning on the unaffected hip at the initial presentation [14]. it was a result of the severity of slippage that developed on the affected hip. Many of the cases (73%) presented with symptoms on the left-sided hip. However, there was no documented identification of this discrepancy.

Many of the cases presented as stable hips, all classified as grade 1. Among these cases, one was identified as an unstable hip. This patient also had a history of hip trauma. The remaining patients presented with chronic symptoms. The results also indicated that the patients had presented symptoms of an unstable hip to either the general practitioner or emergency department, which established symptoms that were related to an affected hip. 

**Figure 2:**
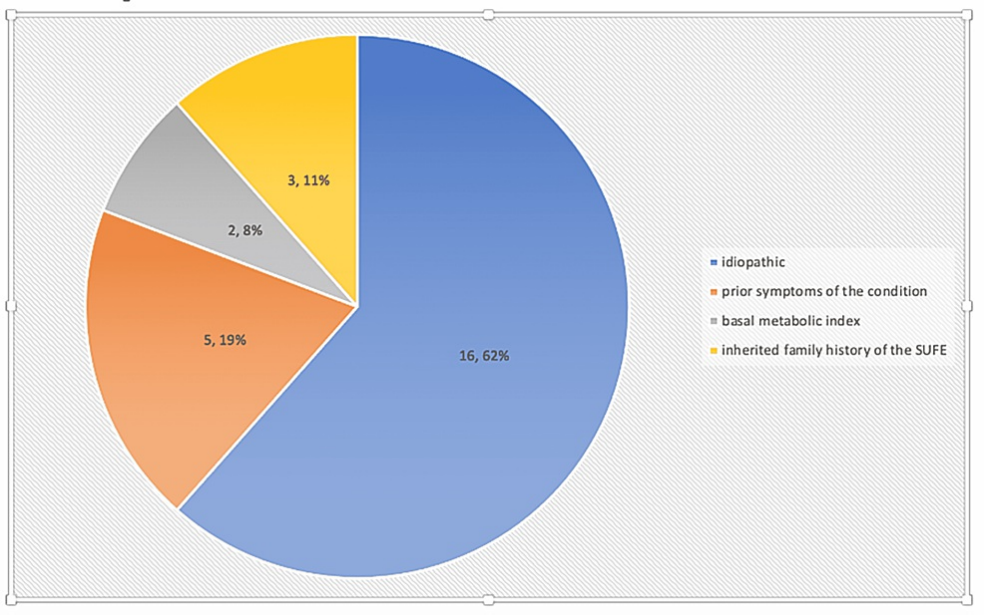
Pie chart depicting the various etiologies of SUFE SUFE: slipped upper femoral epiphysis

In most cases, no definitive underlying causes were observed. Among these cases, 61.5% (n=16) (p<0.05) were idiopathic in nature upon documentation. However, 19% (n=5) (p<0.05) of the cases were shown to be associated with an underlying condition or prior symptoms of the condition, whereas 7.6% (n=2) (p<0.05) had an increased basal metabolic index; 11% (n=3) (p<0.05) of the cases had an inherited family history of the SUFE. Nonetheless, in the patient documents, there was no evidence of data such as basal metabolic index and endocrine profiles (Figure 2).

**Figure 3:**
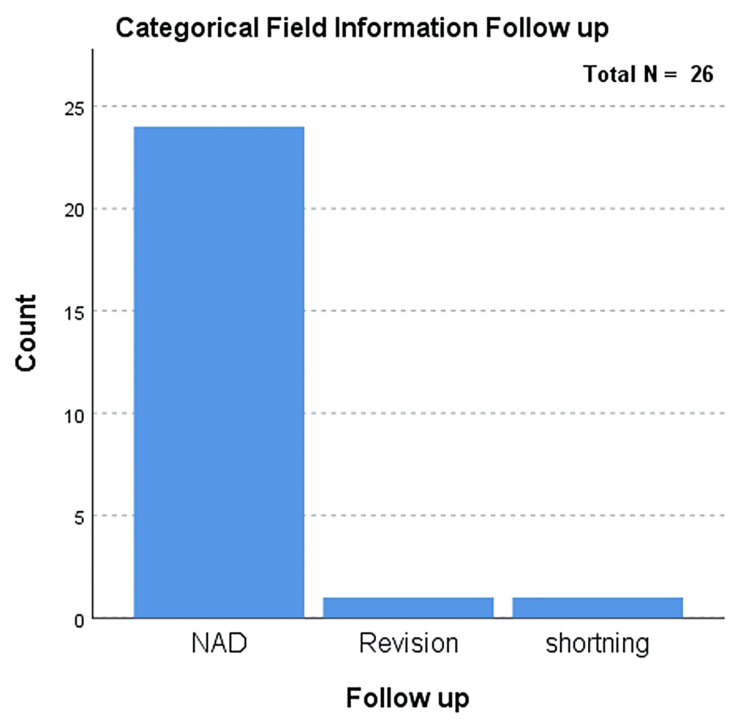
Follow-up after surgical intervention 24 cases had No Abnormality Detected (NAD); one underwent revision and one showed shortening of the affected limb

**Figure 4:**
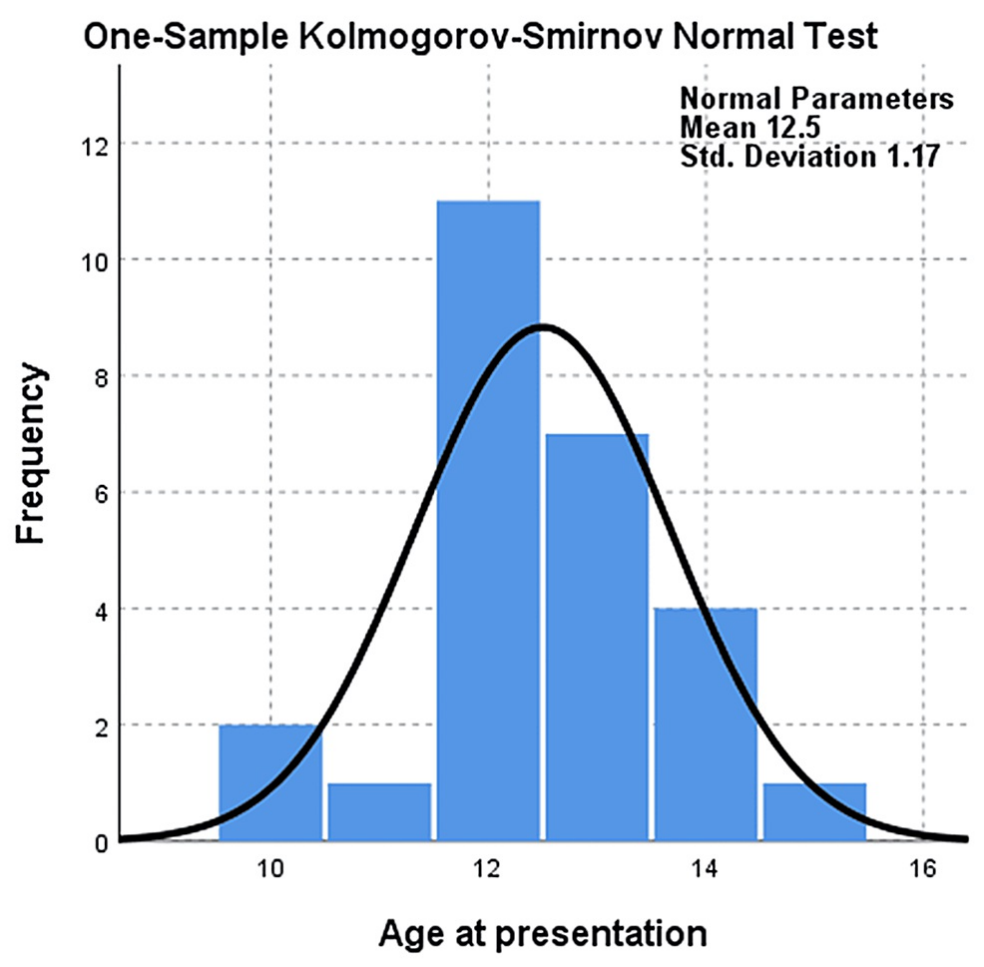
One-sample Kolmogorov-Smirnov normal test for the age of presentation

**Figure 5:**
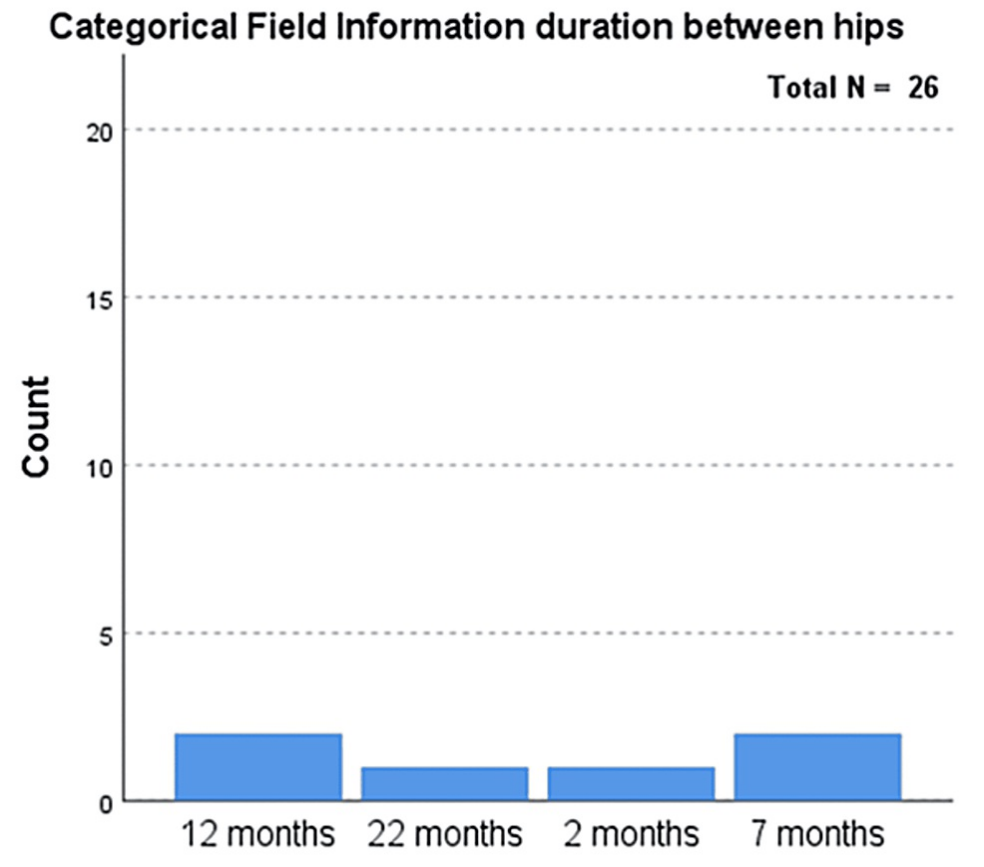
Duration between the interventions for the hips

While investigating the follow-up (mean: two years, SD: 0.5) of the total cases, there was no evidence of post-surgical complications. Only one case required repeat surgery following the initial surgical intervention. Most postoperative complications were well managed due to surgical intervention. Surgery is the first-line treatment in comparison to chemotherapeutics [10]. This case initially presented as a severe hip, making its management challenging. The was another case with an abnormality, that of a shortened limb, which presented as a long-term complication (Figure 3). A comparison between males and females showed a slightly higher frequency of complications in males (n=14) (p=0.556) than in females (n=12) (p=0.556). The age of the participants at the presentation ranged between 10 and 15 years, with an average age of 12.5 years (Figure 4). However, a large portion of the patients (n=11, 42%) had an average age of 12 years (Table 1). The duration between the interventions on hips varied between two and 22 months, with an average of 10.75 months (Figure 5).

**Table 1:** Patient characteristics SUFE: slipped upper femoral epiphysis

Variables	Values (n=26 patients, 52 hips)	P-value
Male/female	14/12	0.556
Average age (range)	12.5 (10-15)	0.002
Idiopathic, n (%)	16 (61.5%)	0.001
Prior symptoms of the condition, n (%)	5 (19%)	0.001
Basal metabolic index, n (%)	2 (7.6%)	0.001
Inherited family history of SUFE, n (%)	3 (11%)	0.001

## Discussion

The age at SUFE presentation in our cohort ranged from eight to 15 years. The mean age at presentation was 12 years. Most of the patients had chronic symptoms of the disease, which highlights the need for raising widespread awareness of the condition. Such awareness is lacking and limited in the public domain. Moreover, the incidence of SUFE has declined in in Ireland recent years [15]. Further investigation into the condition may reveal delayed cases in the current diagnosis. In this regard, much of the emphasis is placed on the details of the SUFE condition with minimal efforts directed toward the aspect of diagnosing the condition [16]. There are several controversies regarding the prophylactic pinning of the unaffected hip in SUFE. Studies have suggested an in situ pinning of a normal hip as a result of a higher slipping rate (2335 times) than that of a normal hip [17]. In contrast, other authors have argued against prophylactic pinning for the unaffected hip due to the exposure of the normal hip to surgical pinning, which could damage the joint, thereby resulting in further exposure of the patient to surgical risks such as infection, fracture, pain, avascular necrosis, and chondrolysis [18]. An analysis of some of the cases revealed devastating deleterious effects of the complications, with some of the cases advocating for non-surgical intervention in managing an unaffected hip. The latter has been performed for the conservative treatment of cases with close follow-up and monitoring [10,14,19].

The current study addressed SUFE cases based on radiological classifications. A meticulous examination was performed with the help of a senior author for the images available on the radiology software at the hospital's records department. However, the hard copies of the X-ray images were not digitalized and were unavailable as self-copies. SUFE was classified based on the Loder classification into unstable and stable hips. The rationale for employing the Loder classification was based on the ability to assess the stability of the patients at the presentation of physis. Moreover, the Loder classification places much emphasis on stability related to the ability to bear the weight of the body [12]. Whether a patient is unstable or stable is based on the ability to bear weight on the affected hip and limb presentation. The inability of a patient to bear weight implies an unstable hip, while the ability to bear weight with or without walking indicates a stable hip.

The majority of the SUFE cases treated at the Sligo hospital were categorized as stable hips, which accounted for 92% of all cases. Only two cases (7.6%) were documented to be unstable hips based on their weight-bearing ability. Other categories of SUFE classification are acute, acute-on-chronic, and chronic. A SUFE is regarded as acute if symptoms are present for less than three weeks, while the acute-on-chronic hip occurs upon exacerbation of the symptoms in an asymptomatic hip, which may result in complications such as chondrolysis, secondary osteoarthritis, and avascular necrosis [18-20]. The current study revealed only two acute cases, both of which were associated with trauma. The remaining 24 cases were classified as chronic, which has been shown to be the most common form of SUFE.

The present study showed that the main cause of SUFE remains unknown since not all cases were investigated for other underlying conditions, especially for the cases presented in the early years; 16 cases (61.5%) were associated with unknown underlying causes. However, 10 (38.5%) cases were shown to have an underlying risk factor or cause. Five cases (19%) showed an antecedent kind of trauma as a leading underlying cause, while two (7.6%) of the cases had a significant family history.

This study has a few limitations, primarily related to its retrospective design. Some of the cases were treated as far back as 15 years ago. Moreover, this study had a small sample size. Hence, the findings of this study cannot be generalized to the wider population.

## Conclusions

This study analyzed cases of SUFE that were treated at Sligo University Hospital over a period of 15 years. Of these cases, two developed complications. Concerning the prophylactic pinning of an unaffected hip, a definitive conclusion could not be reached, as the number of cases was very small. However, we did not find any evidence for the need for prophylactic pinning of the unaffected side or hip. Moreover, it is essential to discuss the chances of the unaffected hip being affected in patients within the first two months following the development of SUFE.
